# Decoding social intentions in human prehensile actions: Insights from a combined kinematics-fMRI study

**DOI:** 10.1371/journal.pone.0184008

**Published:** 2017-08-28

**Authors:** Maria Grazia Di Bono, Chiara Begliomini, Sanja Budisavljevic, Luisa Sartori, Diego Miotto, Raffaella Motta, Umberto Castiello

**Affiliations:** 1 Neuroscience of Movement (NeMo) Laboratory, Department of General Psychology, University of Padova, Padova, Italy; 2 Department of Medicine, University of Padova, Padova, Italy; 3 Centro Interdisciplinare Beniamino Segre, Accademia dei Lincei, Roma, Italy; University of Bologna, ITALY

## Abstract

Consistent evidence suggests that the way we reach and grasp an object is modulated not only by object properties (e.g., size, shape, texture, fragility and weight), but also by the types of intention driving the action, among which the intention to interact with another agent (i.e., social intention). Action observation studies ascribe the neural substrate of this ‘intentional’ component to the putative mirror neuron (pMNS) and the mentalizing (MS) systems. How social intentions are translated into executed actions, however, has yet to be addressed. We conducted a kinematic and a functional Magnetic Resonance Imaging (fMRI) study considering a reach-to-grasp movement performed towards the same object positioned at the same location but with different intentions: passing it to another person (social condition) or putting it on a concave base (individual condition). Kinematics showed that individual and social intentions are characterized by different profiles, with a slower movement at the level of both the reaching (i.e., arm movement) and the grasping (i.e., hand aperture) components. fMRI results showed that: (i) distinct voxel pattern activity for the social and the individual condition are present within the pMNS and the MS during action execution; (ii) decoding accuracies of regions belonging to the pMNS and the MS are correlated, suggesting that these two systems could interact for the generation of appropriate motor commands. Results are discussed in terms of motor simulation and inferential processes as part of a hierarchical generative model for action intention understanding and generation of appropriate motor commands.

## Introduction

The way an object is grasped could depend not only on its physical characteristics like size, shape, texture, fragility and weight [1], but also on the intention driving the action [2, 3]. Using kinematics, several studies have tested whether it is possible to differentiate the reach-to-grasp motor patterns of human agents acting in isolation from those implemented when interacting with others [2]. In particular, the reach-to-grasp movement executed in isolation has been compared with a similar movement executed in the context of a social exchange. In one study [4] participants were asked to reach toward, grasp an object and put it in a concave base (i.e., individual condition), or instead pass it to a co-agent (i.e., social condition). The authors have shown that the two conditions were characterized by distinct kinematic profiles. Specifically, during the reach-to-grasp phase, the maximum hand aperture and the amplitude of peak grip closing velocity were lower for the social than for the single agent condition. In another study [5], it has been demonstrated that an unexpected human social request changes dramatically the kinematics of a pre-planned reach-to-grasp action. At a higher level of abstraction, other reach-to-grasp studies have highlighted specific kinematic patterns for cooperative or competitive behavior with respect to individual conditions [6, 7].

Therefore, the findings that social intentions, defined as intentions accomplished in a context of reciprocal interaction (i.e., the social “why” of the action), can be quantified at the level of the kinematics, contrasts the critical assumptions that social intentions are inaccessible to perception, as things that cannot be seen [8]. Indeed, social intentions seem to have a visible face, which is reflected into different, observable, motor patterns [3].

In contrast to the wealth of behavioral data on this issue, little is known on how the motor system keeps social intentions into account when planning and executing a reach-to-grasp action at a neural level. Consistent evidence suggests that in humans, like in monkeys, reach-to-grasp movements involve a wide fronto-parietal network of interconnected areas [1, 9]. Neuroimaging studies in humans, suggested the hypothesis of two dedicated neural circuits underlying the reaching and the grasping components of the “reach-to-grasp” action [10, 11]: a dorsomedial pathway consisting of superior parietal areas and the dorsal premotor cortex (dPM) for the reach component; a dorsolateral pathway consisting of the anterior intraparietal sulcus (aIPS) and the ventral premotor cortex (vPM) for the grip component [12–15]. This hypothesis, however, has been recently questioned by multi-voxel pattern analysis (MVPA) studies, which provided evidence against a segregation of reaching and grasping circuits [16, 17] and suggested the existence of distinct voxel pattern activity within the same areas of the whole fronto-parietal network, for both reaching and grasping.

To date, it is unclear how the neural circuits underlying the execution of reach-to-grasp movements are modulated by social intentions, though some indirect evidence comes from action observation studies. For instance, the observation of grasping movements performed with social versus individual intents revealed that differential activity within the human putative mirror neuron system (pMNS) [18–20]—which is characterized by an execution component—with specific reference to the inferior frontal gyrus (IFG) and the inferior parietal lobule (IPL), was stronger during the observation of socially intended relative to individual movements [21]. Similarly, areas belonging to the mentalizing system (MS) [22, 23], such as the temporo-parietal junction (TPJ), the medial prefrontal cortex (mPFC) and portions of the middle temporal gyrus (MTG) were more active during the observation of ‘social’ than ‘individual’ grasping movements [21]. Moreover, there is evidence that the areas of the MS (e.g., the mPFC) are more involved when participants have to infer communicative intentions of other individuals (e.g., to offer an apple to another person) rather than individual intentions (e.g., to look at the apple;) [24]. Both the MS and the pMNS can thus be involved in understanding social intentions underlying motor behavior, depending on the goal of the social interaction. Indeed, the pMNS (i.e., the inferior prefrontal-parietal network) seems to be recruited in a more significant way while observing cooperative interactions (e.g., helping each other climb a tree) rather than affective interactions (e.g., establish an affective contact, as for example shaking hands). Differently, the MS (i.e., the mPFC) appeared to be more engaged by affective interactions rather than cooperative ones [25]. It is commonly believed that the pMNS allows to rapidly sense the goal of the perceived other person’s actions, at least for familiar executed actions [26, 27]. The MS, instead, rely on relatively high-level cognitive processes and may allow us to understand other’s goals and beliefs by constructing a Theory of Mind (ToM) that can help us to read other’s mind [28]. Some theorists proposed that the two systems are mutually independent [29], whereas others sustained the idea that the pMNS supports the MS [30–32]. However, the exact nature of the interaction between these two systems is unclear and has become one of the major issues in social neuroscience. One hypothesis is that the pMNS may subserve the low-level (sensorimotor-based) embodied processes that are involved in the decoding of proximal (motor) intentions [20, 33]. In this view, the pMNS may provide a neural route for the rapid, pre-reflective understanding of another person’s intentions. In contrast, the MS might be devoted to higher-level reflective inferences, such as the attribution of complex distal intentions or motives.

In summary, behavioral studies provide consistent evidence that the way we reach and grasp an object is modulated by the intention driving the action. But, how planning and executing a ‘social’ or an ‘individual’ action is encoded at a neural level remains unsolved.

To this purpose, here we performed a (i) behavioral and an (ii) fMRI experiment on the same group of participants, capitalizing on a previously reported paradigm that has the ability to distinguish the nature of intentions underlying the action from their kinematic profile [4]. Participants were asked to reach toward, grasp an object and put it in a concave base (i.e., individual condition), or pass it to a co-agent (i.e., social condition). The behavioral study allowed us to confirm whether social and individual intentions could be distinguished from each other at the level of kinematics. In line with previous findings [4], we expected a delayed and slower kinematic patterning for the social than for the individual condition. For the fMRI study, we used MVPA, based on a linear classifier (Support Vector Machine—SVM) in order to explore: (i) whether and to what extent it is possible to discriminate between social and individual intentions during the execution of a reach-to-grasp action within the pMNS; (ii) whether and to what extent the MS conveys information about social intentions; (iii) whether and how the decoding accuracies from the selected brain areas within the pMNS and the MS are correlated to each other when intention needs to be operationalized into action. The involvement of both the pMNS and the MS in conveying information about the social side of the action has been supported by the aforementioned action observation studies [21, 24, 25]. In line with the previous neuroimaging evidence and with the unifying hierarchical model for motor control and social interaction [34], we hypothesized that the pMNS and the MS should play a crucial role in translating the social intention into an appropriate motor command during action execution.

## Materials and methods

We asked the same group of participants to perform a behavioral and an fMRI experiment. In the behavioral experiment, participants were tested in a kinematics laboratory equipped with six video cameras and the kinematics of the action was recorded during the task performance. In the fMRI experiment, participants were asked to perform the same task (appropriately translated into a paradigm suitable for fMRI data collection) without a simultaneous recording of the kinematics. The order of the experiments was adequately counterbalanced across participants, for controlling potential task-learning effects.

### Ethical statement

Testing was performed in accordance with the ethics approval by the Institutional Review Board at the University of Padua, in line with the Declaration of Helsinki (Sixth revision, 2008). All participants gave informed written consent before participating in the behavioral and the fMRI experiments.

### Behavioral experiment

#### Participants

Twenty-three participants (7 males; mean age: 24.8±4.6, age range: 20–42 years) participated in the experiment. All participants were right-handed as measured by the Edinburgh Handedness Inventory [35]. Participants were recruited through a direct request within the common Institutional spaces. They participated to both the experiments in the period [July 2014—December 2015] and received a monetary reimbursement on completion of the study.

#### Stimuli and procedure

**Fig 1:**
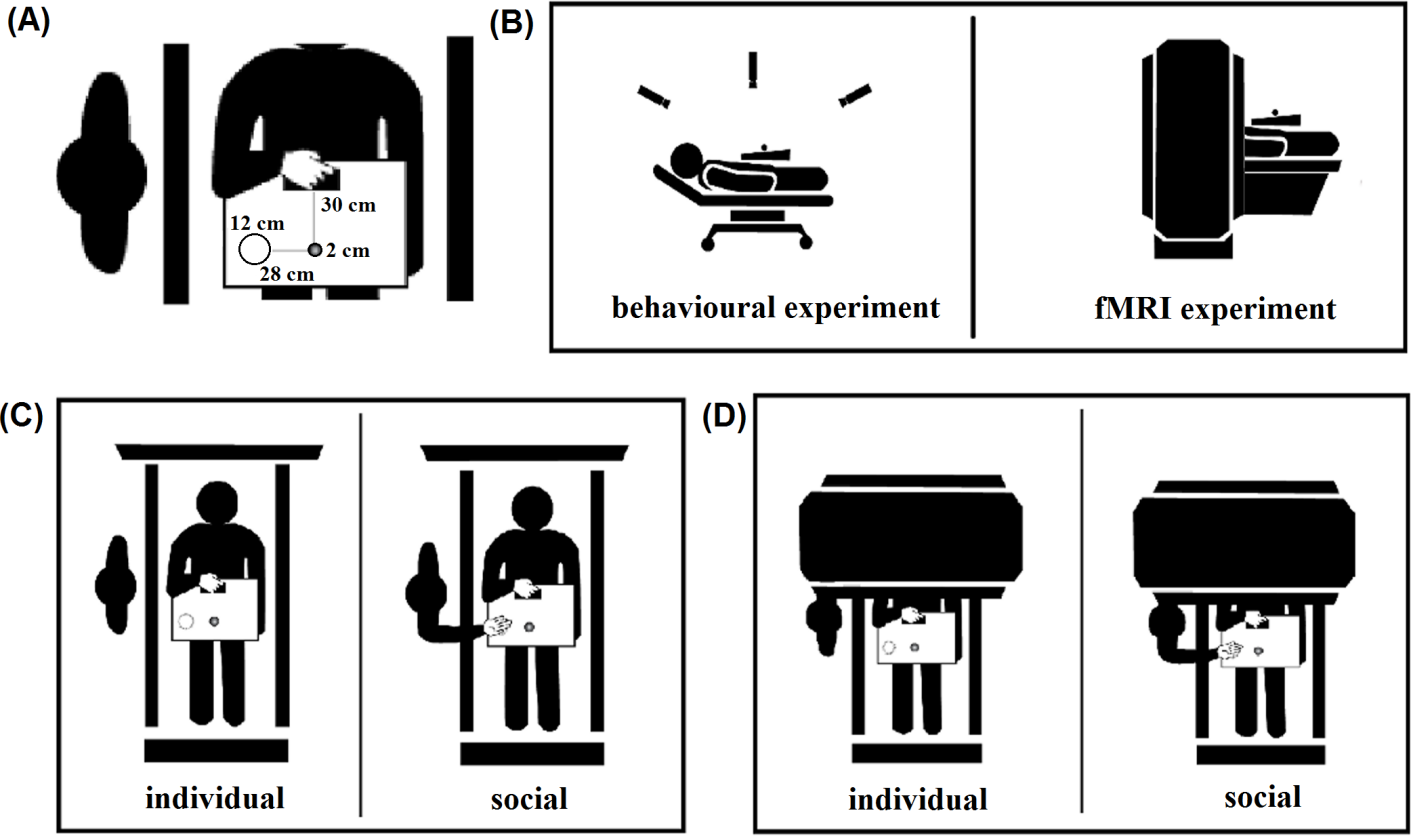
Descriptive example of the experimental setting. Panel (A) shows the distance between the starting position of the hand and the object to be grasped, and the distance of the object (and the co-agent, i.e., the experimenter) from the concave base. Panel (B) shows a descriptive example of the participant position, which was identical in the behavioral and the fMRI experiment. Panel (C) shows an example of the reach-to-grasp movements performed (outside the scanner) in individual and social conditions. Panel (D) shows an example of the reach-to-grasp movements performed (inside the scanner) in individual and social conditions.

The stimulus was an object of spherical shape (2 cm diameter) positioned on a black table in front of the participant at a distance of 30 cm from starting position of the hand, along the midsagittal plane. The concave base (12 cm diameter) was positioned at the participant right side at a 28 cm distance from the target location (see Fig 1, panel A).

Participants were tested individually in a softly lit room. They were laying supine on a bed, in order to control postural effects and matching behavioral and fMRI experimental settings (Fig 1, panel B). Before each trial, the right hand of each participant was resting on a starting pad (a brown velvet cloth 7 × 6 cm) with the index finger and the thumb gently opposed. Participants were asked to start the action after the presentation of a tone (880 Hz/200 ms). The action was divided into a reach-to-grasp and a place phase: the social aspect of the action was manipulated by considering two experimental conditions (Fig 1, panel C): (i) Individual. Each participant was asked to reach towards, grasp the stimulus (reach-to-grasp phase), and put it in a concave base (place phase). (ii) Social. Each participant was asked to reach towards, grasp the stimulus and pass it to another agent whose hand was placed on top of the concave base (place phase), so that the target location was identical to that of the individual condition in both phases. The concave base was equated, in terms of concavity and depth, to the hand of the co-agent as to avoid differences in movement accuracy. The co-agent was seated to the right side of the table with the hand supine resting on the end-pad. The vision of the participants was never occluded during the whole experiment.

Participants were informed about the type of trial (individual/social) by visually inspecting the presence/absence of the hand of the experimenter above the concave base adopted for individual trial. After each trial, the experimenter re-positioned the stimulus on the initial target location. The order of conditions was randomized between participants. Each subject performed 12 trials for each condition.

Analyses were confined to the reach to grasp phase, that is up to the moment the object was grasped. This was done in order to specifically target the ‘intentional’ component. Grasping the same object in order to move it or pass it are both intentional actions. The critical difference is in the intentional component: whereas moving an object contains a purely individual intention, passing an object to another person necessarily involves a social intention, as shown in the published literature.

#### Kinematics recordings

A 3D-Optoelectronic SMART-D system (Bioengineering Technology and Systems, BTS) was used to track the kinematics of the participant’s right upper limb. Three light-weight infrared reflective markers (0.25 mm in diameter; BTS) were taped to the following points: 1) thumb (ulnar side of the nail); 2) index finger (radial side of the nail); and 3) wrist (dorsodistal aspect of the radial styloid process). Six video cameras (sampling rate 140 Hz) detecting the markers were placed in a semicircle at a distance of 1–1.2 m from the bed. The camera position, roll angle, zoom, focus, threshold, and brightness were calibrated and adjusted to optimize marker tracking, followed by static and dynamic calibration. For the static calibration, a 3-axes frame of 5 markers at known distances from each other was placed in the middle of the table. For the dynamic calibration, a 3-marker wand was moved throughout the workspace of interest for 60 s. The spatial resolution of the recording system was 0.3 mm over the field of view. The standard deviation of the reconstruction error was below 0.2 mm for the x-, y-, and z-axes.

#### Data processing

Each trial was individually checked for correct marker identification, and the SMART-D Tracker software package (BTS) was used to provide a 3-D reconstruction of the marker positions as a function of time. Then, data were filtered using a finite impulse response linear filter (transition band = 1 Hz, sharpening variable = 2, cutoff frequency = 10 Hz). Movement onset was defined as the time at which the tangential velocity of the wrist marker crossed a threshold (5 mm/s) and remained above it for longer than 500 ms. For the reach-to-grasp phase, the end of movement was defined as the time at which the hand made contact with the object and quantified as the time at which the hand opening velocity crossed a threshold (−5 mm/s) after reaching its minimum value and remained above it for longer than 500 ms. For both social e individual reach-to-grasp tasks, the following kinematic parameters were extracted for each individual movement using a custom Matlab routine (Matlab 2014b, The 4 Math Works, Natick, MA, USA): the time interval between movement onset and end of movement (Movement Time–Mov_T), the time at which the tangential velocity of the wrist was maximum from movement onset (Time to Peak Wrist Velocity–T_peak_V) and its amplitude (Amplitude of Maximum Peak Velocity–Amp_peak_V), the time at which the acceleration of the wrist was maximum from movement onset (Time to Peak Acceleration–T_peak_A) and its amplitude (Amplitude of Maximum Peak Acceleration–Amp_peak_A), and the time at which the deceleration of the wrist was maximum from movement onset (Time to Peak Deceleration–T_peak_D) and its amplitude(Amplitude of Maximum Peak Deceleration–Amp_peak_D). Furthermore, a grasp-specific measure was assessed, specifically the time at which the distance between the 3D coordinates of the thumb and index finger was maximum, between movement onset and hand contact time (Time to Maximum Grip Aperture–T_max_grip_apert).

#### Statistical analysis

Statistical analysis was performed using JASP software (Version 0.7.5.61; free downloadable at https://jasp-stats.org/download/). Gaussian distribution was confirmed for all kinematic indexes (α-level: *p* < 0.05, all *ps* ≥ 0.09), except for the T_peak_A, and Amp_peak_V (all *ps* ≤ 0.013). We used the Shapiro–Wilk test [36] allowing the use of parametric statistics for the major part of the kinematic indexes. For the two non-normally distributed indexes (i.e., T_peak_A, and Amp_peak_V), we performed non-parametric statistics, using the Wilcoxon signed rank test. The mean value for each kinematic parameter of interest was determined based on 12 individual observations for each participant and then entered into separate paired-samples t-tests for comparing social versus individual reach-to-grasp conditions.

### fMRI experiment

#### Participants

The same participants who took part in the behavioral experiment were tested also in the fMRI experiment.

#### Stimuli, procedure, and experimental design

The stimulus, task and procedures were identical to those adopted for the behavioral experiment; timing and number of trials were modified in order to make the paradigm suitable for fMRI data collection. Participants were laying in the scanner bore with their head tilted at an approximate angle of 30°, supported by a foam wedge permitting direct viewing of the stimulus, and the co-agent arm. The co-agent was standing on the right side of the participant nearby the bore of the scanner and remained present for the entire experimental session (see Fig 1, panel D).

Participants were requested to perform the same action toward the stimulus, which was decomposed into two phases: i) reach-to-grasp phase, in which participants were instructed to reach and grasp the object and keep the hand still on the object until an acoustic go signal indicated the beginning of the subsequent stage; ii) place phase, in which participants were asked to put the grasped object into a concave base (individual condition) or to pass it to a co-agent (social condition), whose hand was on top of the concave base, positioned on the right side of the participant. The two phases were separated by a constant temporal interval of 2 s. Each trial started with a sound (880 Hz/200 ms) delivered by pneumatic MR-compatible headphones, and participants were instructed to start their action toward the stimulus only when the sound was delivered. For each experimental condition, fifty trials were administered, divided into two functional runs. As for the behavioral experiment, the fMRI analyses were confined to the reach-to-grasp phase. Participants were informed about the type of trial (social vs individual) by visually inspecting the presence/absence of the hand of the experimenter above the concave base adopted for individual trial.

The experiment was conducted by using an event-related design, with Inter Stimulus Interval (ISI) varying from 3 to 8 s with a “long exponential” probability distribution [37]. ISIs distribution was fully randomized across trials in each run.

#### Data acquisition and preprocessing

The experiment was carried out on a whole body 1.5 T scanner (Siemens Avanto) equipped with a standard Siemens eight channels coil. Functional images were acquired with a gradient-echo, echo-planar (EPI) T2*-weighted sequence in order to measure blood oxygenation level-dependent (BOLD) contrast throughout the whole brain (37 contiguous axial slices, ascending interleaved sequence, 56 × 64 voxels, 3.5 mm × 3.5 mm × 4.0 mm resolution, FOV = 196 mm × 224 mm, flip angle = 90°, TE = 49 ms). Volumes were acquired continuously for each run with a repetition time (TR) of 3 s; 165 volumes were collected in each single scanning run, resulting in functional runs of 8 min and 15 s duration (16 min and 30 s of acquisition time in all). High-resolution T1-weighted image were acquired for each subject (3D MP-RAGE, 176 axial slices, no interslice gap, data matrix 256 × 256, 1 mm isotropic voxels, TR = 1900 ms, TE = 2.91 ms, flip angle = 15°). Data preprocessing was performed using SPM12 (Statistical Parametric Mapping, Wellcome Institute of Cognitive Neurology, London, UK) implemented in MATLAB 7.5.0 environment (MathWorks, Natick, MA, USA). ArtRepair toolbox (ArtRepair software Package, for SPM12) was adopted to correct for possible images corruption due to signal spikes induced by head motion. Motion correction was carried out by realigning data. Structural images were segmented and subsequently the image of grey matter was co-registered with all the functional images. Structural and functional images were then normalized adopting the template provided by the Montréal Neurological Institute (MNI) implemented in SPM12. No spatial smoothing was applied for classification purposes, because it introduces a certain degree of inter-voxel correlation, and may cause the loss of information useful for separating adjacent but functionally different brain areas.

#### Region of interest specification

To avoid any circularity issue in regions of interest (ROI) selection [38], we did not rely on the functional data but selected four ROIs that were defined purely on anatomical grounds (using the SPM WFU pick atlas toolbox and the Talairach Daemon (TD) anatomical labels (gyral anatomy) atlas, transformed in MNI space; http://www.fil.ion.ucl.ac.uk/spm/ext/#WFU_PickAtlas).

We anatomically selected frontoparietal areas belonging to the pMNS and the MS, focusing on previous neuroimaging findings on action observation [9], showing a BOLD signal increase in areas of the pMNS and the MS (e.g., IFG, IPL, mPFC, and MTG) during social interaction with respect to individual condition. Thus, we bilaterally selected the IFG, the IPL, the mPFC, and the MTG. For a better delineation of the mPFC, we used the Automated Anatomical Labeling (AAL) atlas, selecting the Frontal_Sup_Medial regions.

To test the classifier performance outside of our network of ROIs, we defined two additional non-brain control ROIs in which no BOLD signal was expected and thus no consistent classification performance should be possible (see [39], for a similar methodological procedure). A 5 mm^3^ cubic region was selected within participants’ right ventricle (centroid MNI coordinates: [10, –12, 22]), plus a second 5 mm3 cubic ROI just outside the skull of the brain, near the right visual cortex region (centroid MNI coordinates: [56, –90, –10]). We anticipate that the pattern classification did not reveal any significant decoding in these two areas (i.e., right ventricle: M = .5 ± .01 SEM, t = -.16; outside the brain: M = .49 ± .02 SEM, t = -0.54).

Moreover, we considered the bilateral Posterior Cingulate Cortex (PCC) as a cortical control ROI. This area is particularly interesting because although its activity is associated with social cognitive processing such as self-reflection it seems not to be involved in the kind of social processing at stake here. In a recent review [40], the authors suggested that the PCC activity plays a key role in facing with moral issues regarding ourselves or others, or guilt that may come as a consequence of our actions. Crucially, Johnson and collaborators [41], found a dissociation between the medial prefrontal cortex (mPFC–here considered as part of the mentalising system), and the PCC: the activity of the mPFC was more associated with instrumental or agentic self-reflection (e.g., hopes and aspirations), whereas PCC activity was more associated with experiential self-reflection (e.g., duties and obligations). Furthermore, in their mini-review on the neural bases of mentalizing [22], the authors reported that the mPFC might be concerned with anticipating what a person is going to think and feel and thereby predict what they are going to do. Finally, the role played by the dorsal mPFC in executive inhibition, self-other distinction, prediction under uncertainty, and intention-related processing, has been reviewed in [42], where the authors argued that the involvement of the dorsal mPFC in these processes may explain why it is preferentially activated when people mentalize others' internal states. In light of this literature, we reasoned that the PCC could be a good cortical control ROIs for our study, because it is engaged in social situations that are different from that induced by our social interaction task. We anticipate that decoding results showed that the mean decoding accuracy (averaged across participants) from bilateral Posterior Cingulate Cortex (PCC) was M = 0.52 +/- 0.02 SE. The one tailed t-test (*t*(22) = 1.51, *p* = 0.07) showed that it was not possible to discriminate between individual and social intention from the voxel pattern activity within the PCC control ROI.

#### Classifier decoding

After ROI extraction, the voxel time series were pre-processed through a series of commonly used steps: linear detrending, temporal filtering, and standardization. For every participant, each of the two runs was processed separately. Linear trends in each time series were removed, and a high-pass filter (0.01 Hz) was applied in order to remove low frequency drift in the signal. Then, time series were standardized in order to have zero mean and standard deviation one.

To investigate to what extent each selected ROI was involved in encoding the social intention of the action occurring during the first segment of the action we performed a decoding analysis using the fMRI signal within a temporal window of [3–6 s] from the onset of each trial, which better approximates the BOLD peak of the reach-to-grasp phase of the action.

We used Support Vector Machine (SVM) with linear kernel (using Matlab functions “svmtrain” and “svmclassify”) as multivoxel pattern classifier for discriminating between social vs. individual action intention. For each participant, we trained a linear classifier on the voxels within each selected ROI. The target condition was coded in a way to have a vector Ti ϵ{+1, −1}N, where i refers to the sample and N is the number of samples relative to both conditions in the classification (e.g., N = 100), in which all the samples corresponding to one target condition (i.e., Social) were labeled with +1, whereas all the other samples (i.e., Individual) with −1. The value of the regularization constant C was fixed to 1 (i.e., the default value). Cross-validation was used to estimate the test generalization performance. The SVM classifier was trained on the data set using a modified version of leave-one-out cross-validation, in order to maintain a balanced set of training and test examples. At each step of the cross-validation loop, two samples (one for each condition) were excluded from the training set and used to test generalization performance (see [43, 17]). Classifier accuracy, computed across the entire cross-validation loop on the test set, was used as statistical measures of binary classification.

Previous studies showed that t-test group analysis, with respect to nonparametric randomization tests, is a rather conservative estimate of significant decoding accuracy [39]). Therefore, we conducted a set of one-tailed t-tests, one for each ROI, on the classifier accuracy (against the chance level of 50%) to obtain group statistics regarding the discrimination between the two conditions included in each classification.

All further analyses on the classifier accuracies were restricted to the ROIs from which it was possible to significantly discriminate between social and individual intention. Crucially, in order to investigate the role of the different areas within the pMNS and the MS in distinguishing between individual and social intention, we performed a Repeated Measures Analysis Of Variance (RM-ANOVA) on the mean classifier accuracies, considering the ROI as a within subject factor.

Furthermore, in order to assess whether and to what extent the selected areas cooperate or are independent, we performed a correlation analysis, using Pearson correlation, among the decoding accuracies of the ROIs. We used false discovery rate (FDR) for multiple comparisons correction.

## Results

### Behavioral experiment

**Fig 2:**
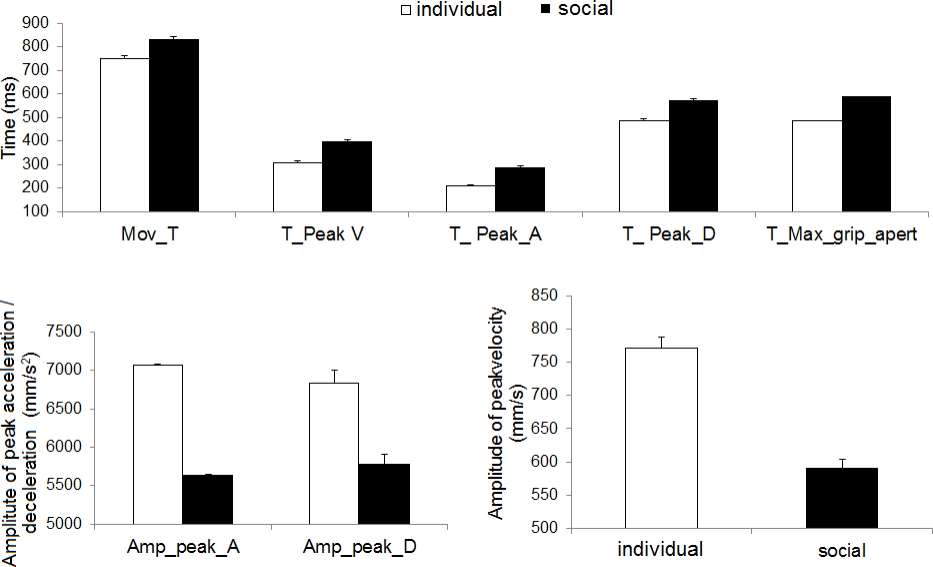
Behavioral results. Movement time and kinematic values showing all the statistically significant differences between the social vs. the individual condition, during the reach-to-grasp phase of the action. Error bars indicate one standard error of the mean. Mov_T = Movement Time, T_peak_V = Time to Peak Wrist Velocity, Amp_peak_V = Amplitude of Maximum Peak Velocity, T_peak_A = Time to Peak Acceleration, Amp_peak_A = Amplitude of Maximum Peak Acceleration, T_peak_D = Time to Peak Deceleration, Amp_peak_D = Amplitude of Maximum Peak Deceleration T_max_grip_apert = Time to Maximum Grip Aperture.

A series of paired t-tests on the kinematics measures (see methods section for details) revealed that the kind of intention driving the action had the ability to modulate kinematics (all *ts* ≥ 9.06; see Table 1 and Fig 2). Specifically, there was an increase in total movement time together with a delayed occurrence of the time to peak velocity, acceleration and deceleration for the social with respect to the individual condition. In addition, also the amplitude for these peaks was lower for the social than the individual condition. Finally, the time of the maximum hand aperture was significantly greater when the action was performed with a social than an individual intent. Single subject kinematic parameters can be found in S1 Table (social condition) and S2 Table (individual condition).

**Table 1:** T statistics on the movement time and kinematic indexes between social and individual conditions during the reach-to-grasp action phase.

***Paired Samples T-Test (Student's T-Test)***							
						**95% Confidence Interval**	
	**t** **df = 22**	**p**	**Mean Difference**	**SE** **Difference**	**Cohen's d**	**Lower**	**Upper**
Mov_T	19.12	< .001	84.13	4.40	799.2	75.00	93.26
T_Peak V	11.31	< .001	90.30	7.99	391.0	73.74	106.87
Amp_peak_A	-10.93	< .001	-1429.30	130.79	5629.2	-1700.54	-1158.07
Amp_peak_D	-9.06	< .001	-1054.74	116.41	5771.0	-1296.16	-813.31
***Paired Samples T-Test (Wilcoxon Signed-Rank Test)***							
						**95% Confidence Interval**	
	**W**	**p**	**Mean Difference**	**SE** **Difference**	**Cohen's d**	**Lower**	**Upper**
T_ Peak_A	276.00	< .001	77.00	6.806	282.7	64.50	90.00
Amp_peak_V	0.00	< .001	-172.45	16.295	581.0	-201.00	-146.00

Mov_T = Movement Time, T_peak_V = Time to Peak Wrist Velocity, Amp_peak_A = Amplitude of Maximum Peak Acceleration, Amp_peak_D = Amplitude of Maximum Peak Deceleration, T_max_grip_apert = Time to Maximum Grip Aperture, T_peak_A = Time to Peak Acceleration, Amp_peak_V = Amplitude of Maximum Peak Velocity.

Altogether these findings suggest that the social action calls for a slower and delayed movement at the level of both the reaching (i.e., arm movement) and the grasping (i.e., hand aperture) components, indexing a more careful movement toward the object, with the intention of positioning it in the co-agent’s hand (i.e., another human agent), with respect to the concave base (i.e., an object).

### fMRI experiment

#### Decoding accuracies

To investigate whether the voxel pattern activity within the considered pMNS and MS could convey information regarding the kind of intention motivating a reach-to-grasp action, we applied a linear SMV classifier to each a priori anatomically selected ROI. Preliminary analyses performed on each ROI and hemisphere did not show evidence on hemispheric differences across all the selected ROIs (see Table 2).

**Table 2:** T statistics on the decoding accuracy from each ROI between left and right hemisphere.

							95% Confidence Interval	
ROI	t	df	p	Mean Difference	SE Difference	Cohen's d	Lower	Upper
IFG (L vs. R)	.41	22	.69	.01	.02	- 4.26	- .04	.06
IPL (L vs. R)	- .69	22	.5	- .01	.02	- 5.66	- .05	.03
MTG (L vs. R)	.03	22	.98	4.348e -4	.02	- 6.45	- .04	.04
mPFC (L vs. R)	- .72	22	.48	- .01	.01	- 7.99	- .04	.02

L = Left hemisphere; R = Right hemisphere.

**Fig 3:**
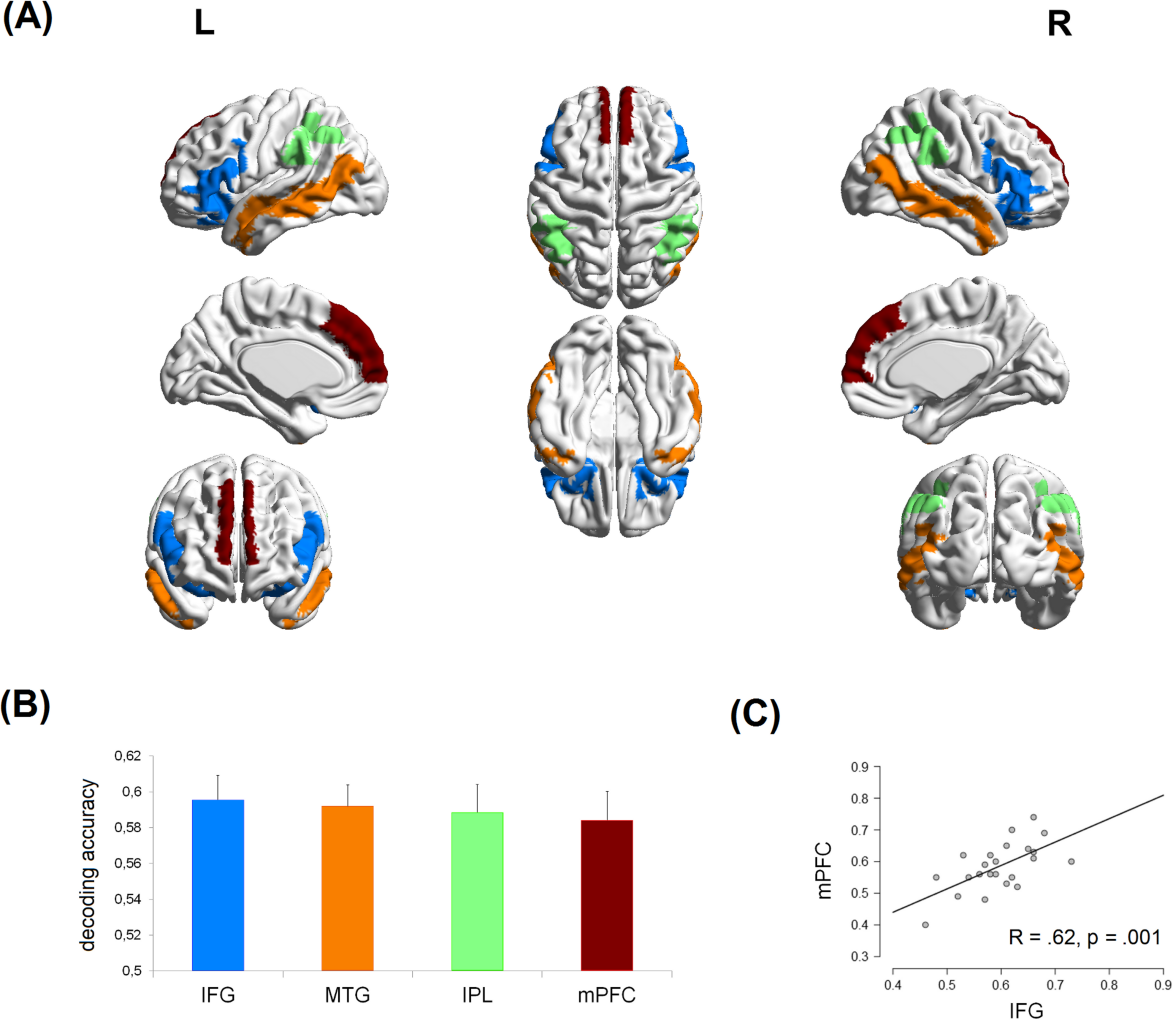
fMRI decoding results. Panel (A) shows regions of interest (ROIs) used in the multivariate classifier analyses, transparently superimposed on top, lateral and mesial view of a standard template using BrainNet Viewer (http://www.nitrc.org/projects/bnv/) [44]. Panel (B) shows the mean linear SVM classification accuracy for discriminating between social and individual conditions of the reach-to grasp action phase as a function of the involved bilateral ROIs. Error bars indicate one standard error of the mean. All decoding accuracies are significantly greater than 0.5 (chance level = 50%; *p* < .001, after FDR correction for multiple comparisons). Panel (C) shows the significant correlation (*p* ≤ .001, after FDR correction for multiple comparisons) between the decoding accuracy of the IFG (pMNS) and that of the mPFC (MS). The greater was the discriminant information about the social intention conveyed by the IFG, the greater was that conveyed by the mPFC. Error bars indicate one standard error of the mean.

We had no a priory hypothesis about a major involvement of one hemisphere over the other in distinguishing between social and individual action. Thus, for each of the selected ROI, we pulled together the voxels belonging to both hemispheres. Classifier results showed that it was possible to decode the social intention of the action from all the selected ROIs (all *ts* ≥ 5.3; see, and Fig 3, panel B, and Table 3), but not, as anticipated, from the control ROIs (*t*s ≤ -.54).

**Table 3:** T statistics (one-tailed one sample t-test) on the decoding accuracy of the selected ROIs.

						95% Confidence Interval	
ROI	t	df	p	Mean Difference	Cohen's d	Lower	Upper
IFG	7.12	22	< .001	.09	1.48	.57	∞
IPL	5.77	22	< .001	.09	1.20	.56	∞
MTG	7.93	22	< .001	.09	1.65	.57	∞
mPFC	5.30	22	< .001	.08	1.12	.56	∞

#### Interaction amongst the mirror and the mentalizing systems

We performed an RM-ANOVA on the mean classifier accuracies, considering the ROI (4 levels) as a within-subjects factor. Results showed no main effects of the ROI (*F*(1.99, 43.76) = .13, *p* = .88, Greenhouse-Geisser corrected for sphericity). Thus, no significant differences among the decoding accuracies of the selected areas emerged.

Finally, we investigated whether and to what extent the decoding accuracies for the selected areas correlated to each other. After FDR correction for multiple comparisons, results showed that the decoding accuracy from the PFC was significantly correlated with that from the IFG (R = .62, p = .001). In summary, correlation results indicate that the mPFC and the IFG do interact. Specifically, the higher was the decoding accuracy from the mPFC, the higher was that from the IFG (see Fig 3, panel C).

## Discussion

We set out to investigate the role of intention (social vs. individual) in modulating the kinematics of the reach-to-grasp action and the neural correlates of these effects. We asked the same group of participants to perform two separate experiments capitalizing on the same paradigm. In the behavioral experiment we focused on the effect of action intention on the kinematics of reaching and grasping movements performed for moving an object from one spatial location to another (i.e., a concave base or the hand of a co-agent). In the fMRI experiment, we investigated whether regions included in the pMNS network could discriminate between social and individual intention, during the reach-to-grasp phase of the action. As a secondary hypothesis, we investigated whether brain areas belonging to the MS could also play a role in encoding the social intention during the execution of the reach-to-grasp action. Finally, we explored the hypothesis of a possible interaction between the two systems.

### Decoding social intentions from kinematics

Kinematical results showed that social intention (i.e., the intention to pass the object to be grasped to the hand of a co-agent) influences action kinematics. Specifically, the reach-to-grasp movement was slower and the kinematics more delayed in the social than the individual condition, as indexed by all the kinematic parameters characterizing both the reaching and the grasping phases. We argue that a slower movement may stand for the need to adopt a more careful and accurate approach when passing the object to another person. Overall, our behavioral results confirm the role of social intention in shaping the kinematics of the action. These results replicate and extend the findings of a previous study [4] to other kinematics measures, and are in line with the idea of a more gentle modulation of hand shaping with the social nature of the action goal [7]. Crucially, here we showed that significant differences emerged between the motor patterns in social and individual conditions, despite the similarity (in terms of experimental setting) of the reach-to-grasp phase of the action for both conditions. Hence, the findings that social intentions can be quantified at the level of action kinematics support the fact that social intentions are accessible to perception [45] and are characterized by distinctive motor patterns [2].

### Decoding social intentions from fMRI data

Classifier results showed that it was possible to discriminate between social and individual conditions from all the selected ROIs of both the pMNS and the MS. In line with the accuracies reported in fMRI studies in the domain of motor control (e.g., [39]) the significant level of accuracy ranged between 58–60%. This low range could be related to the noise present in the fMRI signal and to the fact that no spatial smoothing was performed before the decoding analysis. Spatial smoothing is commonly applied to fMRI data before the conventional univariate analysis (i.e., the General Linear Model–GLM) because it increases the signal-to-noise ratio. When applying multivariate decoding procedures, however, the use of spatial smoothing is not recommended: it could be dangerous, because it might cancel out differences between anatomically adjacent, but functionally distinct, brain areas. Additionally, a possible lack of functional specificity of the selected ROIs could have contributed to the resulting low accuracies. In [46], the authors suggested that decoding from voxel pattern activity within purely anatomical (and large) ROIs could be biased towards a small accuracy, at least in those cases in which only a small percentage of voxel activity is informative for the classifier. Multivariate analysis of fMRI data, however, is more sensitive to distributed coding of information with respect to univariate analysis [47]. The fact that our accuracy values are in line with those reported in other fMRI studies on the motor control, and that null results were found for our non-brain and cortical control ROIs, support the validity of our findings.

On average, the MS and the pMNS were conveying a similar amount of discriminating information. However, different dynamics characterized the correlations among their decoding accuracies.

#### The putative mirror neuron system (pMNS)

Concerning the pMNS, our results provide evidence of a direct involvement of these areas in mediating the social intention underlying the execution of the action: all regions included in the network (i.e., bilateral IFG and IPL) revealed distinct patterns of activity for the social and the individual condition. Only the first phase of the action (i.e., the reach-to-grasp phase) was considered in the analysis. Thus, despite the identical physical experimental setting of the reach-to-grasp phase during social and individual conditions (i.e., the object to be grasped was the same and was located at the same position), the intention underlying the action (i.e., individual vs social component) appeared to be represented by distinct voxel pattern activity. In other words, actions performed with and without a final social aim appeared to be coded differently within the pMNS. This is a novel finding that could answer a controversial question concerning the role of the pMNS in representing the social intentions during the execution of a social action.

The fact that these areas of the pMNS were encoding the action intention through distinct patterns of activity highlights their crucial role in mediating the construction of the final motor code for the appropriate execution of the action. This contribution becomes evident also when considering the difference between the kinematic profile characterizing the two conditions. Our findings are in line with other neuroimaging results investigating the neural correlates of joint action execution, and showing that the coordination of two agents for the achievement of a common goal recruits regions of the pMNS [48–49]. Due to a close link between perception and action provided by the pMNS brain regions, an agent could use simulation for predicting the intentions and consequences of actions performed by the co-agent, in order to adjust his own action planning and then successfully achieve the joint goal. In our experiment, the co-agent was always present and the participants were informed regarding the kind of action to be executed by visually inspecting the presence (i.e., social condition) or the absence (i.e., individual condition) of the co-agent’s hand. We argue that the pMNS could provide an automatic representation of an action based on the visual state of the other, by translating the perceived action (i.e., the hand movement) into motor and somatosensory representation of “how” and “what” the co-agent has done, in order to integrate this information into an appropriate common motor code. This argumentation is in line with the predictive coding account [50] for understanding the function of the mirror system: the most probable causes of an observed action can be inferred by minimizing the prediction error at each level of the cortical hierarchy involved during action observation.

For the execution of the reach-to-grasp action with a social intention, however, the pMNS might be only one component of a hierarchical system for abstracting the intention of the action (i.e., social vs. individual) and integrating it into appropriate motor commands [34, 51].

#### The metalizing system (MS)

The social nature of the intention underlying the reach-to-grasp action appeared to be decoded also within the MS, considered as responsible for the attribution of goals and intentions [51, 52]. During action observation, the MS appeared to become active when observers reflected on the intentionality of an observed action [53–55]. This suggests that MS regions contribute to the analysis of other people’s actions when the viewer decides to reflect upon their goals, intentions and beliefs [30, 51]. But why is the MS recruited for the execution of socially intended movements? Evidence from studying gaze-based social interactions suggests that activity in areas concerned with mentalizing is evident when participants follow the gaze of another person to engage in joint attention [56]. However, no study so far has elucidated the possibility that social information engaging the mentalizing system is conveyed as to influence movement kinematics. Our data on action execution may say something about this issue. We argue that the MS could provide a representation of an action intention by continuously monitoring both the self and the co-agent and that the nature of such process may be the integration of low-level embodied mechanisms within higher level inference-based mentalizing. This may serve to integrate mentalizing kind of information with that coming from low-level embodied processes, into an appropriate common motor code. Indeed, there is empirical evidence on the existence of shared neural circuits for mentalizing about the self and others [57]. Brain regions that are part of the mentalizing network are specifically engaged when we reflect about intentions [55].

Here, we suggest that the MS (in concert with the pMNS) may have the ability to inform regions responsible for shaping action kinematics regarding the ‘social’ intent motivating the action. Another possible role of the MS system might be to monitor, during the on-line control phase, that the social side of action is maintained throughout the movement.

#### Correlation amongst the mirror and the mentalizing systems

In order to test the possible interactions among the areas of the two systems (the pMNS, and the MS) we investigated possible differences and correlations among their decoding accuracies. Our results showed that the selected areas are equally involved in representing the social intention of the action. Further analyses on decoding accuracies showed a different correlation dynamics among the two circuits: the decoding accuracy from the mPFC (MS) was positively correlated to that from the IFG (pMNS): the more informative was the activity pattern encoding action intention within the mPFC, the more informative was that within the IFG. These results confirm the idea that the pMNS and the MS can work in concert [31, 32, 57]. The pMNS might subserve the MS that, in turn, might integrate the representational code provided by the pMNS with its own in order to enrich the motor code with the information concerned with the “what” and the social “why” of an action. Indeed, studies focusing on action observation have shown that the pMNS and the MS do integrate [51]: the mirror system could translate the perceived action into motor and somatosensory representation [58–60] of how and what others do. Such simulated representation could be later interrogated by the MS to ‘reflect’ on why other people act [30]. Action observation and execution could be two faces of the same coin. Indeed, increasing the sensitivity of the fMRI data analysis (i.e., using a single subject analysis and no spatial smoothing), it has been shown that during the observation and execution of hand actions, the classical pMNS areas (i.e., ventral PM cortex and rostral IPL) together with additional brain areas like the MTG, contain “shared voxels” between execution and observation [61].

Hence, our findings are consistent with the idea that motor simulation and mentalizing (i.e., inferential mechanisms) are not mutually exclusive, but play complementary roles in understanding the intentions of other agents around us. Whether the role of the pMNS is subordinate to that of the MS is not clear. One hypothesis is that the pMNS may allow a rapid and pre-reflective understanding of another person’s intentions through sensorimotor processes, whereas the MS may allow higher-level reflective inferences, such as the attribution of complex distal intentions. Although a meta-analysis of functional MRI data suggested that these two systems are relatively independent [32], data from a number of activation or effective connectivity functional MRI studies [57, 62] do not support this view. The two systems may interact and cooperate.

Whether these two systems interact at a horizontal or vertical level within the cortical hierarchy requires further investigation. The unifying framework for motor control and social interaction [34] assumes a vertical hierarchy in the organization of movements with higher levels representing goals and intentions and lower levels representing kinematics and muscle-group selection. In this proposed hierarchical model it is possible to have several representations of the observed action ranging from the low-level kinematics of movement, to the representation of sequence of actions (at the intermediate level), to the task goal representation (i.e., the action intention) at the highest level. This model could be used in a forward and inverse way: the forward model can learn both elementary movements (lower level) and their sequential order (intermediate level) through sensorimotor learning; then, progressively higher levels could learn more abstract representations with the higher level learning task goals (i.e., intentions). Later, in the inverse model, the activation of a high-level goal, or intention, would progressively activate lower levels representations in such a way to generate the appropriate motor commands (at the lower level) to perform the required action. It is not clear, however, at which level of the hierarchical generative model the pMNS and the MS could interact. Neuroimaging studies based on effective connectivity, Diffusion Magnetic Resonance Imaging, and artificial simulations based on hierarchical generative models should help to understand how these two neural systems interact for recognizing the action intention and efficiently generating an appropriate motor command.

Overall, our findings challenge current theories in social cognition, blending motor simulation and inferential processes as part of the same hierarchical generative model for action’s intention understanding and generation of appropriate motor commands. Our findings suggest that the crosstalk between the MS and the pMNS, could be a crucial step in mediating the transition from intention understanding to action execution during social interactions, and could be a valuable insight for understanding which mechanism is impaired in disorders that show deficits in social cognition, such as autism spectrum disorder.

### Strength of the study and steps forward

The present study investigated, for the first time, how the social intention is translated into executed prehensile actions testing the same human subject population at the behavioral and neural level. Furthermore, it is the first study using MVPA for assessing whether it is possible to decode social intention from voxel pattern activity of brain areas within the pMNS and the MS. However, some aspects should be considered for future studies.

Firstly, in our experimental paradigm, our “individual” condition correspond to the "passive observer" condition as in [4], where participants performed the reach-to-grasp action with the intention to place the object in a concave base while the co-agent was visible to their right. Our “social” condition was their “social” condition, where participants performed the reach-to-grasp action with the intent to interact with the co-agent. Becchio and colleagues [4] demonstrated that there is no difference (at the kinematical level) between "single agent" and "passive observer" condition, and claimed that the mere presence of a non- interacting co-agent do not modulate the kinematics of the action, whereas only the interaction between the co-agent and the participant, do influence the action kinematics. However, future studies could consider adding another control condition in order to strengthen the social aspect of the action. For example the use of a robotic hand in a control condition could allow the exploration of whether distinct patterns of voxel activity emerge within the pMNS and the MS when interacting with a robotic vs. a human hand. Indeed, at a behavioral level, Sartori and colleagues [5] investigated the influence of an unexpected social request on the kinematics of a pre-planned action, and showed no kinematics modulation when the perturbation was caused by the robotic agent or by a human agent performing a non-social gesture. Only when the perturbation was characterized by a social request involving a human agent, the kinematics of the action directed toward the target changed.

Furthermore, in order to better disentangle whether the different findings found for ‘to place’ and ‘to give’ are determined by the presence of a different social intention and not only because they are two actions performed to reach two different goals with different accuracy requirements, future studies should include a control condition in which the accuracy for the two goals is equated.

Finally, in our study the visibility of the co-agent was not completely identical during the kinematical and the fMRI experiments. Although the co-agent was always present at the right side of the participant (like in the behavioral experiment), his visibility was reduced by the physical structure of the scanner, due to differences between a setting having an open view (e.g., in kinematical setting), and being in a tube (e.g., in the fMRI setting). However, the co-agent’s arm was always visible by each participant laying supine in the fMRI scanner. We acknowledge this empirical limitation, and propose that future combined kinematics-fMRI studies should decrease the visibility of the co-agent also in the kinematical setting.

## Conclusions

This is the first study investigating through MVPA how the social intention is translated into executed actions, and provides novel insights into the nature of social interactions. Overall, our results showed that social intention shapes the kinematics of the action in terms of a more careful patterning when we interact with another person rather than when performing the same action alone. Critically, we analyzed our kinematical and neuroimaging data only during the reach-to-grasp phase of the action, which allowed us to compare the social and the individual condition in physically identical experimental setting (the object to be grasped was the same and it was positioned at the same location in both conditions). This was done in order to specifically target the nature (individual/social) of the intentional component of the action. Neuroimaging results showed that areas belonging to the pMNS and the MS are involved in encoding different representational codes for the social and the individual intention of the action. In particular the mPCF could interact with the IFG, through a translation of the simulated action code provided by the IFG (i.e., the “what” and the “how” of the action) into an action code equipped with a social meaning (i.e., the social “why” of the action) that could eventually be translated into an appropriate motor command. Our results, even if correlational in nature, fit well with the hierarchical unifying framework for motor control and social interactions of Wolpert and colleagues [34]. Future studies using structural, functional and effective connectivity, as well as simulations based on hierarchical generative models should help to understand how these two neural systems interact for recognizing the action intention and for efficiently generating an appropriate motor command.

To conclude, examining how the decoding of intentions is modulated by the degree to which healthy humans perceive themselves as participants of an ongoing interaction is of crucial importance in social cognition. Our results may help to shed light on the putatively complementary roles of mirror and mentalizing networks in situations that better approximate those encountered in daily life.

## Supporting information

S1 Table Single subject kinematic parameters in social condition. (PDF)

S2 Table Single subject kinematic parameters in individual condition. (PDF)
