# Supplementary material for: Decoding social intentions in human prehensile actions: Insights from a combined kinematics-fMRI study
Source: PLoS One. 2017 Aug 28;12(8):e0184008. doi: 10.1371/journal.pone.0184008 (PMC5573299; doi:10.1371/journal.pone.0184008)
Supplement: S1 Table — (PDF) [file pone.0184008.s001.pdf]

Supporting information

S1 Table. Single subject kinematic parameters in social condition.

Subject	Mov_T	T_Peak V	T_ Peak_A	T_ Peak_D	Amp_peak_V	Amp_peak_A	Amp_peak_D	T_max_grip_apert
1	852	400	302	601	702	6211	6094	631
2	818	396	279	557	592	5761	5721	612
3	800	387	275	560	643	5871	5764	576
4	798	377	265	541	612	4345	5213	631
5	845	412	312	559	618	6002	6212	603
6	922	437	319	622	588	5435	5576	598
7	794	355	248	513	657	5876	5861	509
8	854	417	307	600	562	5567	5987	598
9	847	423	318	600	582	6012	5768	617
10	786	346	235	531	598	5324	5256	641
11	841	413	306	587	581	4567	5934	609
12	823	411	309	567	602	5234	5004	602
13	881	456	341	618	587	5878	5902	561
14	900	432	309	645	456	5523	5873	631
15	799	387	254	523	581	5621	5467	600
16	764	339	231	562	608	6543	6123	584
17	824	400	287	571	561	5231	5854	598
18	879	378	265	603	434	5198	6231	612
19	842	441	326	632	603	6021	5888	548
20	834	387	296	554	678	6988	6732	555
21	841	418	311	568	512	5728	5912	554
22	900	425	302	546	612	5112	5076	552
23	756	341	254	498	621	5682	5567	578
