# Supplementary material for: Decoding social intentions in human prehensile actions: Insights from a combined kinematics-fMRI study
Source: PLoS One. 2017 Aug 28;12(8):e0184008. doi: 10.1371/journal.pone.0184008 (PMC5573299; doi:10.1371/journal.pone.0184008)
Supplement: S2 Table — (PDF) [file pone.0184008.s002.pdf]

Supporting information

S2 Table. Single subject kinematic parameters in individual condition.

Subject	Mov_T	T_Peak V	T_ Peak_A	T_ Peak_D	Amp_peak_V	Amp_peak_A	Amp_peak_D	T_max_grip_apert
1	768	254	207	453	865	8210	8123	448
2	734	328	199	516	701	7021	7122	509
3	698	329	206	458	756	7256	7127	446
4	695	300	211	503	771	7234	6212	510
5	765	299	225	482	768	7345	6543	508
6	856	342	222	482	674	5889	6123	500
7	711	318	197	527	805	6934	6424	523
8	789	316	218	512	775	7213	6612	498
9	741	310	222	502	781	7213	7123	511
10	701	288	205	520	812	8213	6423	523
11	801	298	235	512	745	6574	6734	498
12	754	291	198	520	801	6621	6145	512
13	786	360	243	490	786	7543	7498	501
14	851	352	218	511	691	6234	6545	521
15	705	287	189	502	704	7127	5234	462
16	681	298	188	496	801	7421	7325	438
17	718	341	201	509	687	6122	7428	522
18	821	317	231	500	876	6134	6734	541
19	754	238	142	384	892	7865	7777	402
20	735	309	219	437	954	8675	8453	456
21	781	322	214	521	678	6847	6712	509
22	778	303	238	467	723	5679	5734	481
	642	301	217	458	725	7234	7123	458
